# Aligning Priorities of Surgeons and Patients Regarding Obesity and Joint Replacement: Recommendations of the Advisory Committee Codesigning Solutions Regarding Obesity and Arthroplasty Consensus Working Group

**DOI:** 10.1016/j.artd.2026.102127

**Published:** 2026-08-27

**Authors:** K. Godziuk, L. Rubinett, C. Van’t Haaff, E. Bohm, H. Chaudhry, A. Cohen-Rosenblum, B. Ricciardi, N.J. Giori

**Affiliations:** a Department of Physical Therapy and Rehabilitation Science, School of Medicine, University of California, San Francisco, CA, USA; b Department of Agricultural, Food and Nutritional Science, Faculty of Agricultural, Life and Environmental Sciences, University of Alberta, Edmonton, AB, Canada; c Patient-Partner and Co-Lead, Austin, TX, USA; d Patient-Partner and Co-Lead, Vancouver, BC, Canada; e Department of Surgery, Max Rady College of Medicine, University of Manitoba, Winnipeg, MB, Canada; f Orthopaedic Innovation Centre, Winnipeg, MB, Canada; g Division of Orthopaedic Surgery, Sunnybrook Holland Orthopaedic and Arthritic Centre, University of Toronto, Toronto, ON, Canada; h Department of Orthopedic Surgery, NYU Grossman School of Medicine, New York, NY, USA; i Department of Orthopaedic Surgery, University of Rochester, Rochester, NY, USA; j Department of Orthopaedic Surgery, School of Medicine, Stanford University, Stanford, CA, USA; k Chief of Orthopedic Surgery, VA Palo Alto Health Care System, Palo Alto, CA, USA

**Keywords:** Obesity, Body mass index, Total joint arthroplasty, Clinical practice guidelines, Optimization

## Abstract

Elective total joint arthroplasty (TJA) in patients with a high body mass index (BMI) or obesity is a complex and nuanced topic. Patients’ and surgeons’ perspectives on how to address the challenges related to TJA in high BMI patients may differ, highlighting an imperative to include perspectives from both groups in strategizing approaches to improve care. Hip and knee arthroplasty surgeons and patients with high BMI and osteoarthritis were included in a consensus working group (Advisory Committee Co-dEsigning SolutionS regarding Obesity and Arthroplasty [ACCESS-OA]) to identify areas of agreement and disagreement on priorities to improve TJA care for patients with a high BMI. In this white paper, we report on the results of this working group. Based on consensus scoring, the working group prioritized addressing uncertainties in (1) presurgical patient optimization, (2) surgeon education and training, and (3) shared decision-making. Advancing research and practice in these priority areas could have important clinical impact.

## Introduction

Many complex ethical issues arise when providing surgical care to patients, and competing interests are common. One area of particular challenge in the realm of total joint arthroplasty (TJA, referring to total hip and knee arthroplasty) involves caring for patients with obesity or a high body mass index (BMI). For brevity, we interchange these terms [1]; however, obesity should not be defined based on BMI alone, and all individuals with a BMI ≥30 kg/m^2^ do not have obesity [2,3].

Arthroplasty surgeons aim to provide high quality care, minimize complications, improve efficiency and throughput, and maintain the financial viability of their practices [4]. Patients with obesity and osteoarthritis (OA) aim to achieve pain relief and improved function and quality of life. They hope to receive high quality care and minimize their risk of complications but commonly encounter barriers to receiving TJA care [5]. Often these patients are repeatedly rejected by multiple surgeons and experience weight bias (ie, negative attitudes, stereotypes, or moral judgment about body size) and stigma (ie, differential treatment due to weight or body size) in their journey through the health care system [6].

One method that has been used by arthroplasty surgeons to reduce the risk of complications after TJA has been to implement a BMI cutoff for surgical eligibility. This approach was influenced by associations between BMI and TJA complications, leading to a 2013 statement from the American Association of Hip and Knee Surgeons suggesting that TJA should be delayed in patients with a BMI ≥40 kg/m^2^ for weight loss before surgery [7]. The application of these BMI eligibility criteria were common, with varying BMI cut points (eg, BMI <35, <40, <50, or none) between difference practices, hospitals, and individual surgeons [[8], [9], [10], [11]]. Ongoing debate arose regarding concerns of restricting access to effective OA care [12], and the American Association of Hip and Knee Surgeons updated their guidelines in 2023 to advise against the use of strict BMI criteria for TJA eligibility [13]. However, the factors that led to these eligibility criteria still exist and are complex [10,14], and thus this practice may continue and be difficult to change. Information and education may help shift perspectives and safely improve practice.

## Problem statement

TJA in patients with a high BMI can be a more complex procedure influenced by surgeon experience, reimbursement limitations, patient factors, medical comorbidities, and other healthcare aspects. However, the current approach to TJA eligibility considerations based on BMI criteria or presurgical weight loss expectations creates disparities in access to care and potential negative repercussions on patient health outcomes. New perspectives to address this issue are needed. Both arthroplasty surgeons and patients with a high BMI and OA want high-quality arthroplasty care with minimization of risk, but how best to advance clinical care and what to focus on in research and practice improvements is uncertain.

## Proposed solution

We brought together surgeons and patients to explore varying priorities and solutions in a consensus codesign working group called ACCESS-OA [Advisory Committee Co-dEsigning SolutionS regarding Obesity and Arthroplasty]. This participatory action consortium of invited arthroplasty surgeons and patients with lived experience of obesity and advanced OA came together to share and discuss perspectives on this topic and identify key problem areas to focus on for solutions to improve care. In this white paper, we report on the findings of this group.

### Engagement and meeting processes of the ACCESS-OA working group

We sought to (1) identify problem areas for potential care advancement by engaging both patients and surgeons in a collaborative consensus working group and (2) identify the most important of these areas to focus efforts to improve care by having patients and surgeons assign priority to these problem areas.

We used participatory action research methods [15], including organizing a working group of surgeons and patients, conducting qualitative content analyses of meeting discussions and a cross-sectional prioritization survey. Participatory action research involves community members (eg, patients with lived experience), not just academics or clinicians, in codesigning the research, framing questions, collecting data, and analyzing results. Participatory action research methods can address complicated or unresolved health care challenges by bringing diverse perspectives into decision-making [16]. They involve genuine engagement and input from key service partners (ie, those delivering and receiving health care) [17,18]. Foundational principles of collaborative community-based participatory research [16] underpinned our approach to identify shareholder-driven priorities for addressing challenges with obesity and TJA [19]. Ethics approval was provided by the Research Ethics Board at the University of Alberta.

A codesign framework from Ramos et al. [20] was adopted as a model for the ACCESS-OA working group proceedings [[20], [21], [22]]. All planning was led by a core leadership team that included 2 patients with relevant lived experience (L.R. and C.V.H.), an arthroplasty surgeon (N.G.), and a mixed methods researcher with expertise in obesity (K.G.). The planning meeting process was informed by engagement theories [23] and strategies to dismantle power imbalances and build working relationships between shareholder groups. All participants provided informed consent.

ACCESS-OA was conducted as a virtual consensus meeting. Participant recruitment and two 4-hour meetings were held in 2023. Participants were purposefully invited, using targeted approaches to recruit patients and surgeons through connections of core team members, prior research involvement, and public invitations shared through knowledge partners, including the Arthritis Society, Obesity Canada, and the Obesity Action Coalition. We included patients with lived experience of a BMI ≥35 kg/m^2^ and knee and/or hip OA who received, considered, or were denied hip and/or knee arthroplasty, and orthopaedic surgeons who specialized in hip and knee arthroplasty and had experience with issues related to obesity and TJA. Individuals with a BMI ≥35 kg/m^2^ are a patient group who are more likely to experience restrictions in access to TJA compared to peers with a BMI <35 kg/m^2^ [10,13]. Some participating surgeons enforced variable use or cut points of BMI eligibility criteria as a policy or due to prior experiences. Others never enforced a BMI criterion. Meeting attendance was capped at 30 participants, with equal distribution of surgeons and patients to allow for feasibility of effective discussions within the videoconference platform. We invited individuals from varied settings and with unique or opposing opinions to gather diverse perspectives on this topic. Individuals who responded to the invitation completed a brief online expression of interest to ensure intentions were focused on system-level solutions.

The meeting agenda included a review of current evidence, shared presentations on patient and surgeon viewpoints, and small and large mixed-group discussions to generate perspectives on problem areas of knowledge gaps and practice uncertainties. The meetings were led by a facilitator with experience in participatory methods and encouraging differing viewpoints. To mitigate power imbalances [22], all participants used first names only to reduce influence of professional titles, and options for verbal and written contributions to discussions were encouraged from attendees. Disagreement was welcomed as part of the consensus-building process. All meeting proceedings were recorded. Verbal discussions and written chat were amalgamated into a summary transcript and anonymized for analysis.

### Analysis and survey

The working group discussion transcript from meeting 1 was evaluated using qualitative content analysis. Two members of the research team (S.R. and K.G.) independently coded and paraphrased the content from the meeting transcript to identify preliminary problem areas that were discussed regarding care for patients with obesity and advanced OA. Then, they met to discuss and refine these identified problem areas first among themselves and then with the core leadership team (L.R., C.V.H., N.G., and K.G.). Any disagreements were resolved through group discussion and consensus, allowing surgeon and patient leaders to review and clarify results prior to finalization. The result was a list of problem areas, knowledge gaps, and practice uncertainties that arose in the codesign discussions of meeting 1 and were ready for priority voting.

The agreed problem areas were then listed in an online survey built in REDCap [24]. This survey was sent to working group members, following a delphi-inspired voting process to determine consensus regarding priority problem areas where efforts could be focused to improve patient care. On the survey, each problem area was accompanied by a 5-point Likert scale for the survey participants to express their opinion on the priority that should be placed on addressing this problem to improve care. The Likert scale went from 0 (low priority) to 4 (high priority). We pre-selected thresholds indicating high- and low-priority agreement based on prior consensus evidence [25,26]. Consensus high priority was determined by ≥ 70% of participants scoring a thematic area with a 3 or 4 on the Likert scale. Consensus low priority was determined by ≥ 30% of individuals scoring a thematic area with 0 to 1 on the Likert scale. Topic areas that received high priority votes ≥70% were discussed in-depth at the second meeting, identifying projects under each priority problem area for the working group to advance in ongoing efforts.

### Working group results

Forty-one invited individuals expressed interest in participating (23 surgeons, 18 patients), with 31 available on proposed dates and providing consent. Twenty-nine attended and participated in the meetings (Table 1). Fifteen were surgeons (11 men), and 14 were patients (all women).

**Table 1:** Description of N = 29 individuals who participated in ACCESS-OA working group meetings.

Participant characteristic	Surgeons (n = 15)	Patients (n = 14)
Sex, male	11 (73)	0 (0)
Country of residence		
Canada	7 (47)	10 (71)
United States	8 (53)	4 (29)
Age categories, years		
Under 40	5 (33)	0 (0)
40-55	5 (33)	4 (29)
56-75	5 (33)	10 (71)
Meeting attendance		
Both 1 and 2	10 (67)	14 (100)
Meeting 1 only	3 (20)	0 (0)
Meeting 2 only	2 (13)	0 (0)

Values are presented as *n* (%).

**Figure 1:**
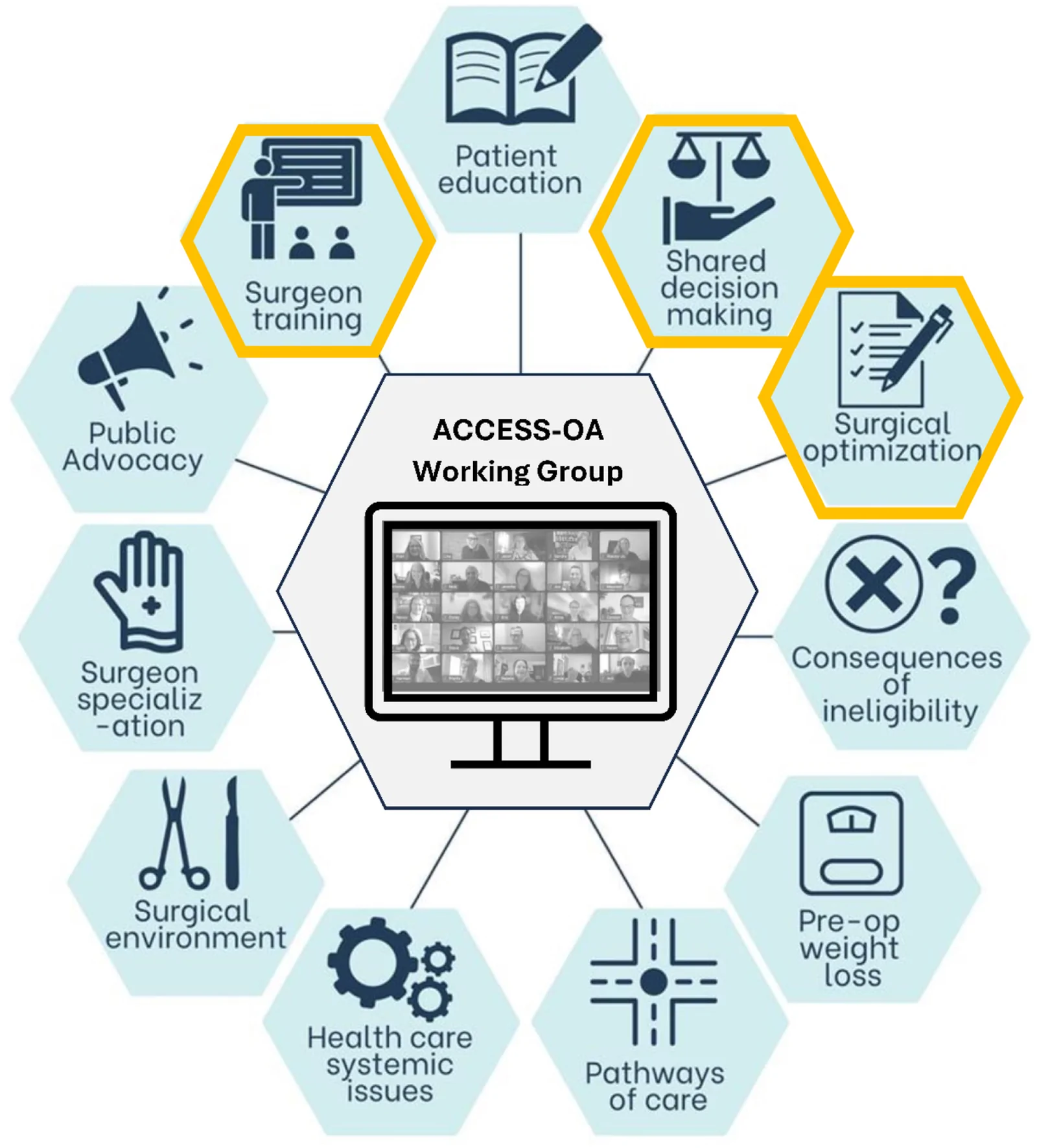
Eleven problem areas of knowledge gaps and practice uncertainties regarding obesity and arthroplasty identified from analysis of working group meeting discussions. Details describing each area are outlined in Table 2. Orange borders signify the 3 areas that were voted as high priority to address by both surgeons and patients together. Voting results are provided in Figures 2 and 3.

**Figure 2:**
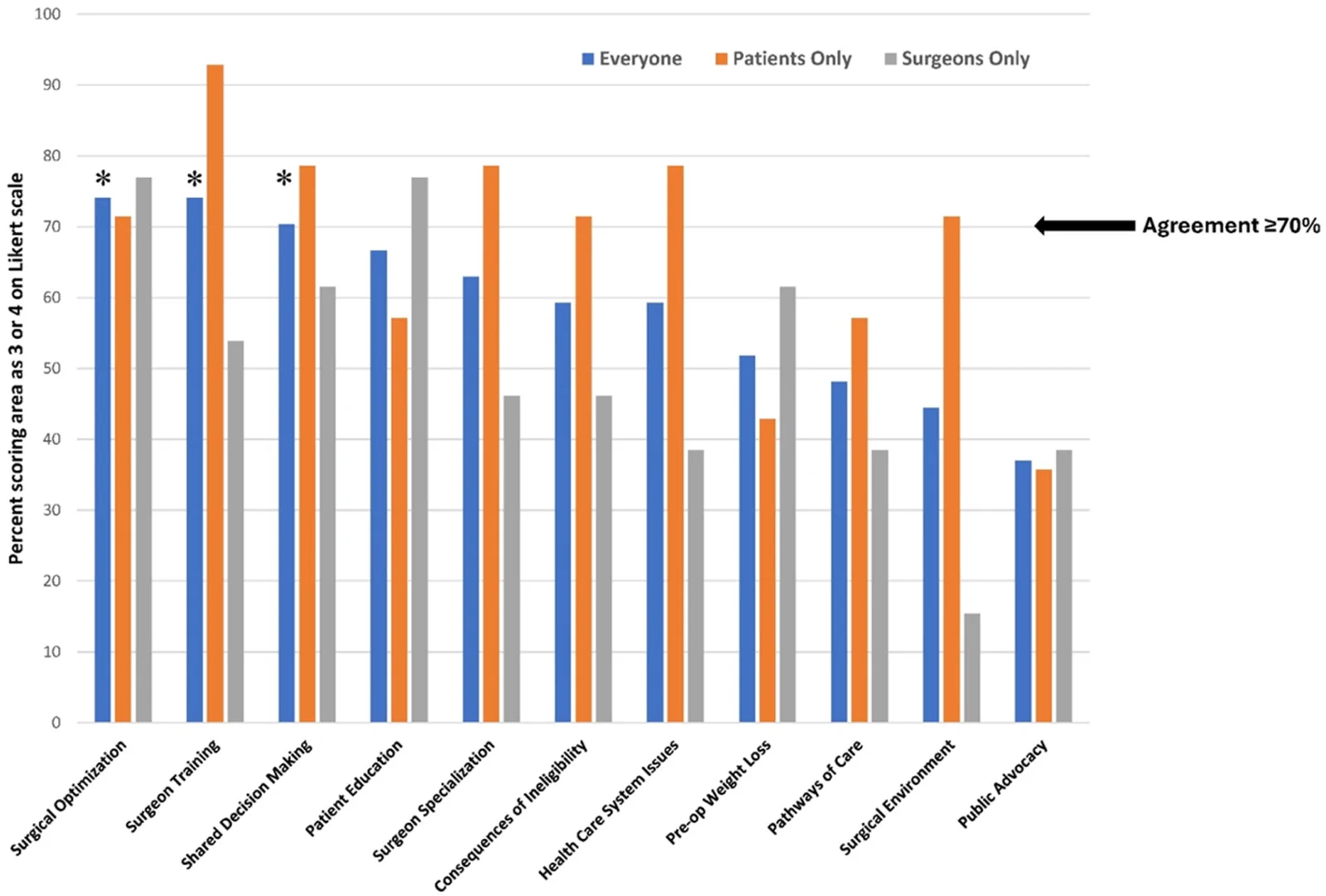
Problem areas scored by meeting participants as medium-high (3) or high (4) priority on Likert scale; 3 areas met consensus agreement threshold of ≥70% for high priority to address based on total group votes∗.

**Figure 3:**
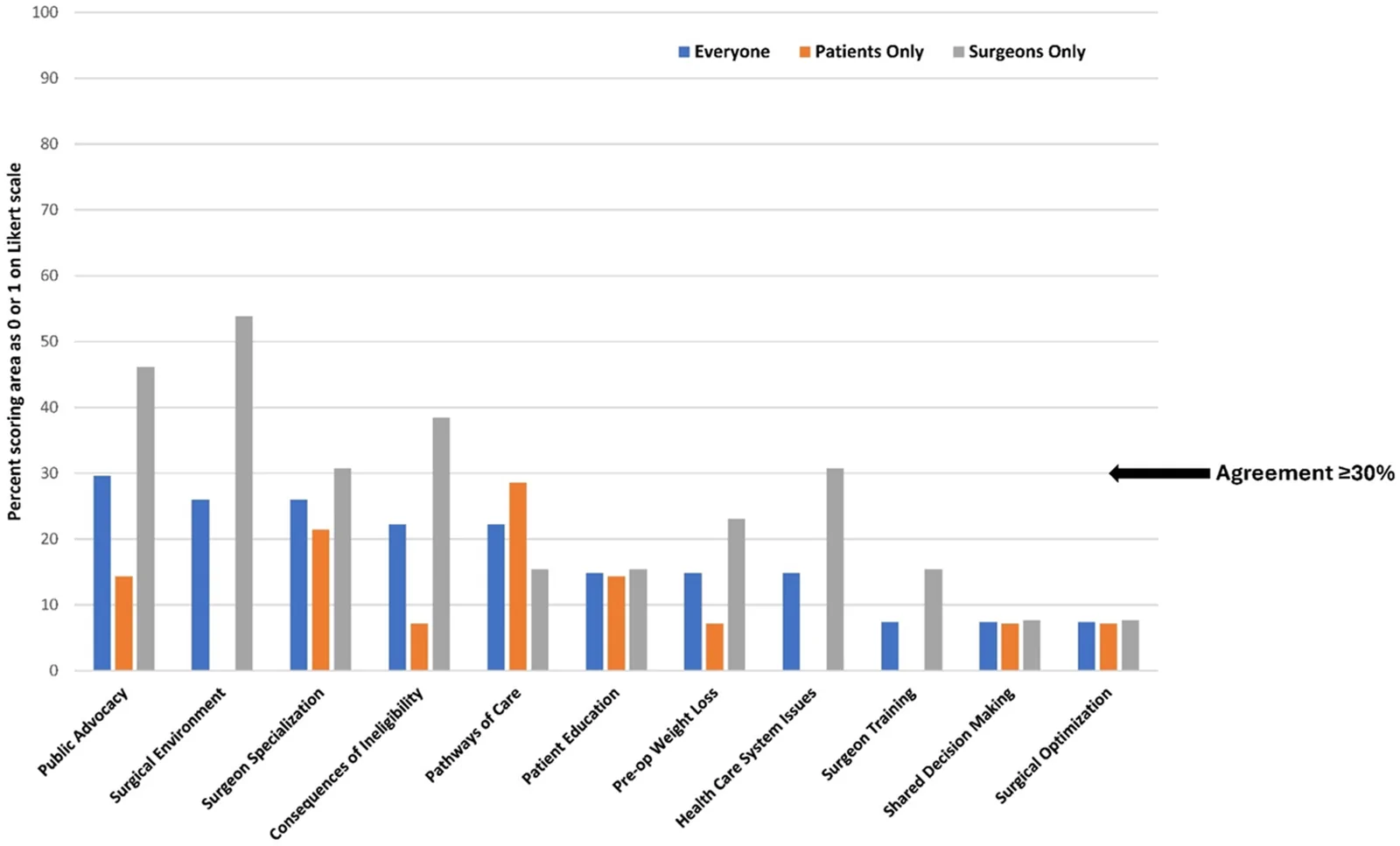
Problem areas scored by meeting participants as a low (0) or medium-low (1) priority on Likert scale; no areas met consensus agreement threshold of ≥30% for low priority based on total group vote.

Eleven problem areas relevant to TJA and obesity were identified upon review of the meeting transcripts, summarized in Figure 1 with details in Table 2. Twenty-seven participants ranked the importance of these eleven problem areas after the first meeting. Based on consensus voting as a group, three high-priority problem areas that should be focused on with future research or quality improvement processes to facilitate improved patient care were identified (Fig. 2). These three problem areas met consensus priority agreement threshold of ≥70% for the group: (1) optimizing patients for surgery, (2) surgeon education and training, and (3) shared decision-making (Fig. 2). No problem areas were identified as consensus low priority by the overall group (Fig. 3).

**Table 2:** Description of eleven problem areas of knowledge gaps and practice uncertainties related to obesity and arthroplasty resulting from ACCESS-OA meeting discussions.

Thematic description of problem area	Examples of paraphrased perspectives of surgeons and patients derived from meeting discussion transcripts
Patient education and self-advocacy What education or resources would be beneficial for patients?	For example, a patient-advocacy guidebook or decision-support tool to help when consulting with a surgeon; information about the surgical procedure, complications, and technical difficulty to support patient decision-making; patient preferences for treatment options if they are unable to have joint replacement
Surgeon training and education What education, training, or support do surgeons need to improve care delivery for their patients?	For example, education around issues of weight bias and compassionate communication skills; training in obesity science to understand difficulty of weight loss and limitations of BMI for surgical eligibility decisions; education regarding technical aspects of performing arthroplasty in high BMI patients
Shared decision-making What tools, resources, or new research evidence would improve the shared decision-making process between surgeons and patients? How can we improve individualized surgical risk assessment?	For example, individual risk calculators specific to this patient population that consider factors beyond BMI; develop and test a decision-tree or algorithm to help patients and surgeons together to evaluate risk of surgery vs risk of waiting or weight loss; assessment guide or committee-based approaches for unbiased evaluation of surgical eligibility; considering patients' overall health status
Surgical optimization in high BMI patients How can we best prepare high BMI patients for a positive surgical outcome? What is effective for risk management and improving patient recovery after surgery?	For example, implications of pre-surgical weight loss; optimal window or timing for surgery; comparative effectiveness of different existing models of preop care; effectiveness of strength training and nutrition rather than weight loss
Consequences of surgical ineligibility What are the second-order consequences of patients being ineligible for surgery? How can we better understand and evaluate the risks of not having surgery?	For example, implications on chronic pain, disability, work productivity, quality of life; comparative evaluation of risk of surgery vs risk of delay
Preoperative weight loss What are the benefits and risks of presurgical weight loss?	For example, impacts on surgical complications and technical aspects of surgery; risk of malnutrition and loss of muscle mass/quality; improvements in symptoms and decisions to proceed to surgery; consequences of weight regain after presurgical weight loss; patient interest and uptake of medications for obesity treatment; access to resources to assist with weight management
Pathways of care What pathways of care are most appropriate and beneficial for this patient population?	For example, develop and evaluate standardized preoperative care pathways for patients with high BMI; compare multidisciplinary vs specialized care; pathways to other resources for preoperative and postoperative support; availability of referral to obesity treatment if patient chooses; how to reduce variability in care for this patient population
Health care system issues What other factors in the health care system support continued use of BMI restrictions for joint replacement? How common are these issues, and how can they be addressed?	For example, anesthesia limits, operating room equipment and support, hospital policies, reimbursement limitations, risk adjustment and incentivization for surgeons, professional backlash or penalties for complications
Surgical environment How can we address technical issues and make surgery easier and consistent for surgeons, and safe as possible for high BMI patients?	For example, equipment and techniques which could help with the technical aspects of surgery; proctors to help surgeons with difficult cases; surgeon training; innovation; surgeon ability to assess and communicate their limitations or comfort level
Surgical specialization and centralization Can we improve patient access to care and outcomes by establishing specialized centers or surgeon specialists?	For example, bariatric orthopaedic specialization, centers of excellence for high BMI joint replacement; centralized guide or list of surgeons; patient support to navigate to right surgeon; cost-effectiveness of more direct referral; addressing inconsistencies in care; consequences of centralization for patients in rural areas
Public advocacy What broader education or advocacy is needed, and how could it influence access to care and surgical capacity?	For example, education for primary care physicians regarding joint replacement eligibility; addressing limitations in access to allied health professionals [ie, nutrition, exercise, physical therapy]; advocacy and lobbying to government and insurers for adjusted surgical reimbursement models

When voting responses were stratified by patients and surgeons, priority rankings differed. Surgeons voted only 2/11 problem areas as high priority (patient education and optimizing patients for surgery). Patients voted 7/11 problem areas as high priority (surgeon education and training, shared decision-making, optimizing patients for surgery, consequences of ineligibility, health care system issues, surgical environment, and surgeon specialization). The only overlapping high-priority problem area was preoperative patient optimization. Patients did not identify any problem areas as low priority (0/11), whereas surgeons voted 5/11 as low priority (consequences of ineligibility, healthcare system factors, surgical environment, surgical specialization, and public advocacy).

### Discussion of results

Preoperative patient optimization was the only overlapping high-priority area common to both patients and surgeons. Surgeon education and training and shared decision-making also met group consensus priority thresholds. Other problem areas were identified as high priority by one group, but not the other. For example, surgeons dismissed nearly half the identified issues as low priority, while patients felt all were important. This illustrates the misalignment in urgency and disparate viewpoints between surgeons and patients.

Improving presurgical optimization was a high-priority item identified by both surgeons and patients. However, there may be a disconnect in what this means to both parties. A common misconception among surgeons is that patients with obesity have less interest or are less engaged in their overall health and preparedness for surgery than patients without obesity [10]. This is weight bias. A common point of discussion for patients in our working group was that they cared about minimizing their surgical risk, but wanted personalized risk assessments beyond BMI, and evidence-based information on whether weight loss would meaningfully improve their surgical outcomes. Patients also wanted surgeons to know that many of them had spent much of their adult lives trying to lose weight. Currently, there are many different types of pre-operative programs that are implemented at different medical centers and surgeon offices which aim to optimize patients for surgery. These programs vary in their treatment approaches (eg, dietitian consult, obesity medications, bariatrics referral) and outcome targets, with some aiming for comorbidity improvement (eg, glycemic control) and others targeting weight reduction [27,28]. Future research should focus on objectively evaluating these preoperative patient optimization programs to identify approaches that most effectively balance risk reduction with maintaining access to quality arthroplasty care. The best program may or may not involve weight loss for patients with a high BMI, as a growing body of literature is casting doubt on the effectiveness of weight loss on reducing TJA complication risk [[29], [30], [31], [32], [33]]. Current recommendations for weight loss may be an oversimplified and mistargeted approach for preoperative risk reduction. There is potential for negative long term repercussions of rapid, unsustainable, and/or unsupervised presurgical weight loss, including risk for malnutrition, sarcopenia, and frailty, which are understudied in TJA and obesity.

Surgeon education received a consensus high-priority vote from the group as a whole and from patients, but not from surgeons. This is a very interesting disconnect that should prompt some thought and reflection by surgeons. Among the arthroplasty community in general, there is discussion that TJA in patients with a BMI ≥40 kg/m^2^ should be conducted at specialty centers to ensure safe care [34]. Surgeon volume [35], experience operating on patients with a high BMI [36], and operative time [37] (ie, reflecting surgeon expertise and resources) may influence TJA complication risk in this population [38]. However, specialized centers may create additional socioeconomic-related access barriers for patients with obesity by requiring them to travel outside of their community for TJA. Discussion of specialized centers and pathways of care were brought forward in our co-design interactions, but did not rank as a high priority by patients or surgeons. Further exploration of this issue may be warranted, including guiding patients to find a surgeon with demonstrated success in safely conducting TJA in patients with obesity, and ensuring that future surgeons are adequately trained to operate on this patient population.

Shared decision making was the final high-priority problem area identified by the group as a whole and by patients, but interestingly not by surgeons alone. Current processes may not be widely accepted as sufficient, primarily by patients. This is another important disconnect. Shared decision-making is a part of the informed consent process, requiring a conversation between the surgeon and patient to discuss individualized surgical risk evaluation, risks and benefits of the procedure, patient values and preferences, and mutual agreement on goals and priorities [39]. Patients want a clear and complete informed consent process, including autonomy and engagement in decision-making, compassionate care, and unbiased communication of risk related to their comorbidities, including obesity [40]. Surgeons may need strategies for effective conversations in time-limited clinical encounters. Future research focused on this step of the informed consent process will help improve care for this patient population.

The involvement of only women as patients in our working group is notable and is a limitation, as the experiences and perspectives of men with obesity and advanced OA are not included. While this may reflect greater interest from women in engaging in advocacy networks [41,42], it also supports evidence that women are more likely to have potential unmet needs for OA care [43]. Women are more frequently undertreated for OA [43], encounter more barriers in access to TJA [5] and more weight-based discrimination, compared to men [44,45]. This is concerning and suggests a need for targeted policy approaches for women in OA care and TJA eligibility [46]. A predominant gender dynamic also exists during real-world TJA surgical consultations, as arthroplasty surgeons are overwhelmingly men [47]. This gendered influence in the TJA clinical encounter may influence the misalignment in priorities between surgeons and patients in our findings. Data suggesting that surgeon and patient gender discordance can negatively influence surgical outcomes [48] highlights the relevance of this area for further investigation in obesity and TJA.

Both patients and surgeons were actively engaged in this participatory action process and demonstrated a high level of commitment, attending two 4-hour virtual meetings and participating in voting. The continued involvement of both surgeons and patients in post-meeting project steering teams to address high priority areas suggests that participants perceive positive experiences and value with participatory action research.

Participatory action research is a qualitative method that often includes a small sample size to achieve meaningful engagement with participants and gather their experiential knowledge [15,16,49]. This method can uncover unique perceptions and insights into a complex topic [50]. We purposefully selected surgeons to be on the panel who practiced in various settings and had different thoughts on how to manage patients with advanced OA and obesity to mitigate this limitation. We did not collect information on race/ethnicity or socioeconomic status of participants, or target participant invitations by sex or gender. Our working group was restricted in number to enable effective conversations on the video platform. The qualitative methods we used do not aim to be representative of all patient and surgeon perspectives [50,51]. Our goal was not to establish generalizable findings but rather to identify purposefully sampled shareholder-informed priority areas to guide future research and policy discussions. The variety of experiences and perspectives among patients and surgeons led to meaningful and enriched discussion in our meetings. Our results provide a unique perspective on how participatory research methods can help align the views of both surgeons and patients to inform better patient outcomes and access to care in TJA.

## Future direction and long-term focus

The misalignment of prioritization of other problem areas related to obesity and arthroplasty must be explored further, as they represent incongruent and potentially opposing surgeon and patient perspectives that may result in diminished medical care. Misaligned priority areas suggest that improving communication between patients and surgeons may increase understanding across the doctor–patient relationship. For example, patients may be unaware of the stress felt by surgeons regarding poor patient outcomes [10], including the potential for negative hospital and individual surgeon ratings as well as financial penalties for complications such as infection and readmission [52]. Surgeons may choose to avoid operating on high BMI patients to reduce this emotional and professional burden. Conversely, surgeons may be unaware or dismissive of the physical and emotional stressors carried by patients with painful end-stage OA, including having to seek multiple surgeon opinions [53], being repeatedly rejected, and experiencing obesity stigma and weight bias through these encounters [54,55]. In addition, patients raised concerns about the consequences of surgical ineligibility on employment, relationships, and financial stability. Efforts to educate both surgeons and patients on each others’ experiences and perspectives may help improve patient–surgeon communication and shared decision-making regarding patient eligibility for TJA. Notably, divergent perspectives between patients and physicians on care delivery are not unique to the field of hip and knee arthroplasty [56]. Singular perspectives in research (ie, initiated by surgeons and addressing their priorities) may not effectively answer questions that are relevant from the patient perspective. Future research regarding obesity and arthroplasty should engage and consider both patient and surgeon perspectives in the design and planning phases along with interpretation of findings.

The working group processes fostered a heightened awareness among participants of the differing priorities held by surgeons and patients. This recognition appeared to mitigate some of the tension that typically arises with misaligned perspectives. Although this effect was observed in the limited context of the meeting, it highlights the potential value of engagement as a model for improving alignment and mutual understanding in the surgeon–patient relationship.

## Recommendations

High-quality research and practice improvements that address uncertainties in presurgical optimization, surgeon training gaps, and enhanced shared decision-making processes should be prioritized to improve arthroplasty care for patients with obesity. These three priority problem areas were identified as important by the consensus working group, though only presurgical optimization was prioritized by both patients and surgeons when considered as separate groups. Many divergent perspectives and priorities between surgeons and patients were identified as well. These highlight the importance of engaging viewpoints from both groups. For surgeons, understanding how patient perspectives may differ from their own should help to foster effective communication and thus improve patient care.

These recommendations from our consensus-informed priority framework provide a foundation to guide future efforts to improve TJA care delivery and surgical outcomes for patients with obesity. This project was not designed to identify specific practice modifications or guidelines, but rather to inform a priority framework. Future work from our ACCESS-OA team will identify, implement and evaluate actionable improvements to address surgeon training gaps, patient optimization, and shared decision-making. Our findings emphasize the value of engaging both surgeons and patients to inform improvements for safe and equitable access to quality arthroplasty care.

## Conflicts of interest

Kristine Godziuk received research grant funding from Canadian Institutes for Health Research; and serves as a Committee member for Osteoarthritis Research Society International, Orthopaedic Research Society, and American College of Sports Medicine. Eric Bohm is a consultant for Stryker; receives honoraria for speakers bureau activities or paid presentations for Stryker; serves as a Board member for International Society of Arthroplasty Registries – past president, Canadian Arthroplasty Society – past president, Chair, Canadian Joint Replacement Registry Advisory Committee; receives receipt of equipment, materials, drugs, medical writing, gifts, or other services from Institutional research and/or fellowship support from Zimmer Biomet, Stryker, DePuy, Smith and Nephew, Hip Innovation Technology. Anna Cohen-Rosenblum is a consultant for Zimmer-Biomet, Smith and Nephew; serves as a Board member for AAOS, AAHKS, RJOS, SICOT; and is a Deputy Editor. Benjamin Ricciardi receives grants or contracts National Institutes of Health (R01 [coinvestigator]); receives honoraria for speakers bureau activities or paid presentations for American Board of Orthopaedic Surgery; serves on the editorial board for Clinical Orthopaedics and Related Research, Arthroplasty Today, The Knee. All other authors declare no potential conflicts of interest.

For full disclosure statements refer to https://doi.org/10.1016/j.artd.2026.102127.

## Funding

Funding for this project was provided by the Canadian Institutes of Health Research and fellowship awards from Alberta Innovates and Obesity Canada.

## CRediT authorship contribution statement

**K. Godziuk:** Writing – review & editing, Writing – original draft, Project administration, Methodology, Investigation, Funding acquisition, Formal analysis, Conceptualization. **L. Rubinett:** Writing – review & editing, Validation, Methodology, Investigation. **C. Van’t Haaff:** Writing – review & editing, Validation, Methodology, Investigation. **E. Bohm:** Writing – review & editing, Validation, Methodology, Investigation. **H. Chaudhry:** Writing – review & editing, Validation, Methodology, Investigation. **A. Cohen-Rosenblum:** Writing – review & editing, Validation, Methodology, Investigation. **B. Ricciardi:** Writing – review & editing, Validation, Methodology, Investigation. **N.J. Giori:** Writing – original draft, Validation, Methodology, Investigation, Formal analysis, Conceptualization.

## Appendix A Supplementary data

Conflict of Interest Statement for Cohen-Rosenblum

Conflict of Interest Statement for Chaudhry

Conflict of Interest Statement for Ricciardi

Conflict of Interest Statement for Van’t Haaff

Conflict of Interest Statement for Godziuk

Conflict of Interest Statement for Rubinett

Conflict of Interest Statement for Giori

Conflict of Interest Statement for Bohm
