# Supplementary material for: Aligning Priorities of Surgeons and Patients Regarding Obesity and Joint Replacement: Recommendations of the Advisory Committee Codesigning Solutions Regarding Obesity and Arthroplasty Consensus Working Group
Source: Arthroplast Today. 2026 Aug 27;41:102127. doi: 10.1016/j.artd.2026.102127 (PMC13543976; doi:10.1016/j.artd.2026.102127)
Supplement: Supplementary file 2 [file mmc2.pdf]

INDIVIDUAL CONFLICT OF INTEREST STATEMENT

American Association of Hip and Knee Surgeons

(Adopted from the American Academy of Orthopaedic Surgeons disclosure statement)

The following form **must be filled out completely and submitted by each author (example, 6 authors, 6 forms). All items require a response. If there is no relevant disclosure for a given item, enter "*None.*"**

Aligning priorities of surgeons and patients regarding obesity and joint replacement: Recommendations of the ACCESS-OA consensus working group

Manuscript Title

1. Royalties from a company or supplier (The following conflicts were disclosed) None

2. Speakers bureau/paid presentations for a company or supplier (The following conflicts were disclosed) None

3A. Paid employee for a company or supplier (The following conflicts were disclosed) None

3B. Paid consultant for a company or supplier (The following conflicts were disclosed) None

3C. Unpaid consultants for a company or supplier (The following conflicts were disclosed) None

4. Stock or stock options in a company or supplier (The following conflicts were disclosed) None

5. Research support from a company or supplier as a Principal Investigator (The following conflicts were disclosed) None

6. Other financial or material support from a company or supplier (The following conflicts were disclosed) None

7. Royalties, financial or material support from publishers (The following conflicts were disclosed) None

8. Medical/Orthopaedic publications editorial/governing board (The following conflicts were disclosed) None

9. Board member/committee appointments for a society (The following conflicts were disclosed) None

Each author must sign AND print or type his/her name, date and submit a separate form

In addition, one BLINDED Conflict of Interest form (no author names used) should be submitted per manuscript with all author disclosures.

Harman Chaudhry	[signature]	August 10, 2026
Author Name (Print or Type)	Author Signature	Date
