# Supplementary material for: Aligning Priorities of Surgeons and Patients Regarding Obesity and Joint Replacement: Recommendations of the Advisory Committee Codesigning Solutions Regarding Obesity and Arthroplasty Consensus Working Group
Source: Arthroplast Today. 2026 Aug 27;41:102127. doi: 10.1016/j.artd.2026.102127 (PMC13543976; doi:10.1016/j.artd.2026.102127)
Supplement: Supplementary file 8 [file mmc8.pdf]

INDIVIDUAL CONFLICT OF INTEREST STATEMENT

American Association of Hip and Knee Surgeons

(Adopted from the American Academy of Orthopaedic Surgeons disclosure statement)

The following form **must be filled out completely and submitted by each author (example, 6 authors, 6 forms). All items require a response. If there is no relevant disclosure for a given item, enter "*None.*"**

Aligning priorities of surgeons and patients regarding obesity and joint replacement: Recommendations of the ACCESS-OA consensus working group

Manuscript Title

1. Royalties from a company or supplier (The following conflicts were disclosed) None

2. Speakers bureau/paid presentations for a company or supplier (The following conflicts were disclosed) Stryker

3A. Paid employee for a company or supplier (The following conflicts were disclosed) None

3B. Paid consultant for a company or supplier (The following conflicts were disclosed) Stryker

3C. Unpaid consultants for a company or supplier (The following conflicts were disclosed) None

4. Stock or stock options in a company or supplier (The following conflicts were disclosed) None

5. Research support from a company or supplier as a Principal Investigator (The following conflicts were disclosed) None

6. Other financial or material support from a company or supplier (The following conflicts were disclosed) Institutional research and/or fellowship support from Zimmer Biomet, Stryker, DePuy, Smith and Nephew, Hip Innovation Technology

7. Royalties, financial or material support from publishers (The following conflicts were disclosed) None

8. Medical/Orthopaedic publications editorial/governing board (The following conflicts were disclosed) None

9. Board member/committee appointments for a society (The following conflicts were disclosed) International Society of Arthroplasty Registries – past president; Canadian Arthroplasty Society – past president; Chair, Canadian Joint Replacement Registry Advisory Committee

Each author must sign AND print or type his/her name, date and submit a separate form

In addition, one BLINDED Conflict of Interest form (no author names used) should be submitted per manuscript with all author disclosures.

Eric Bohm	EBohm	August 10, 2026
Author Name (Print or Type)	Author Signature	Date
